# Effects of a multidisciplinary management program on symptom burden and medication adherence in heart failure patients with comorbidities: A randomized controlled trial

**DOI:** 10.1186/s12912-022-01130-7

**Published:** 2022-12-07

**Authors:** Parvin Ghobadi, Mohammad Gholami, Shirin Hasanvand, Tahereh Toulabi, Nasrolah Moradifar, Mehdi Birjandi

**Affiliations:** 1 Student Research Committee, Lorestan University of Medical Sciences, Khorramabad, Iran; 2 Social Determinants of Health Research Center, School of Nursing and Midwifery, Lorestan University of Medical Sciences, Khorramabad, Iran; 3 Cardiovascular Research Center, School of Nursing and Midwifery, Lorestan University of Medical Sciences, Khorramabad, Iran; 4 Interventional Cardiology Fellowship, Department of Cardiology, School of Medicine, Lorestan University of Medical Sciences, Khorramabad, Iran; 5 Nutritional Health Research Center, School of Health and Nutrition, Lorestan University of Medical Sciences, Khorramabad, Iran

**Keywords:** Comorbidities, Disease management, Heart failure, Medication adherence, Symptom burden

## Abstract

**Background:**

Comorbidities in heart failure (HF) are a complex clinical challenge. There is little data on the benefits of multidisciplinary postdischarge management programs in such patients. This study aimed to examine the effects of a multidisciplinary management program (MMP) on symptom burden and medication adherence in HF patients with comorbidities.

**Methods:**

In this clinical trial study, 94 HF patients with comorbidities were assigned to intervention (*n* = 47) and control (*n* = 47) groups by the stratified-random method. The intervention group underwent MMP supervised by a nurse for two months after discharge, including multi-professional visits, telephone follow-ups, and an educational booklet. Medication adherence and symptom burden were assessed using Morisky Medication Adherence Scale (MMAS) and Edmonton Symptom Assessment Scale (ESAS), respectively, on three occasions: Before discharge, six weeks, and eight weeks after discharge.

**Results:**

Both groups almost matched at the baseline, and the most frequent comorbidities included myocardial infarction (MI), hypertension, peptic ulcer, and depression, respectively. The interactive effect of time in groups showed that mean changes in total scores of symptom burden and medication adherence were significantly different (*P* < 0.001) at other time points. A significant increase in medication adherence (*P* < 0.001) and a significant reduction in the burden of all symptoms were observed in the intervention group compared to the control group from Time 1 to Time 3.

**Conclusions:**

The MMP (targeting comorbidity) is a promising strategy for managing symptoms and medication adherence in HF patients with comorbidities.

**Supplementary Information:**

The online version contains supplementary material available at 10.1186/s12912-022-01130-7.

## Background

Heart failure (HF) often coexists with cardiac or non-cardiac comorbidities [1]. A study reported common comorbidities in HF patients as hypertension (91%), iron deficiency (64%), chronic kidney disease (CKD) (61%), anemia (56%), diabetes mellitus (50%), arterial fibrillation (AF) (49%), and chronic obstructive pulmonary disease (COPD) (26%) [2]. Comorbidities increase the risk of poor health outcomes. Approximately 61% of hospital admissions are attributable to comorbidities that occur within 15 days of discharge [3]. Moreover, higher comorbidity levels predict a lower quality of life (QoL) [4].

Comorbidity is a significant issue that complicates HF management, including symptom management and medication adherence [5]. By polypharmacy, comorbidity is a crucial risk factor for medication non-adherence [6] and adverse drug reactions [7]. Although medication adherence as a critical self-care behavior is necessary for maintaining physiological stability, reducing cardio-circulatory burden, reducing symptom burden, and increasing survival [8], it is not observed by 50% to 62% of HF patients [9]. Poor adherence to evidence-based medications is associated with a fourfold increase in HF-related hospitalization [10], increased health care costs, morbidity and mortality [11], and exacerbation or more significant HF symptom burden [8]. Non-adherence is a multidimensional problem [12]. In addition to comorbidities, medication-specific factors such as adverse drug reactions [7], also aspects related to the disease, the patient, and the healthcare system, like health disparities, inadequate health literacy, and depression, play a role in medication non-adherence [13]. The most critical reasons for medication non-adherence in HF patients are forgetfulness, fatigue, orthopnea, or lack of symptom relief [14].

HF patients experience debilitating symptoms with different severities [15]. Challenges such as symptom clusters, inadequate symptom-related knowledge, poor assessment skills, and lack of trust in health professionals’ expertise and support hinder the management of these complex symptoms [16]. According to the middle-range theory of unpleasant symptoms, certain physiological factors, including comorbidities [17], and psychological factors are associated with symptom burden and management [18]. A higher burden of symptoms impairs daily living activities, QoL, and self-management behaviors in HF patients [19].

The direct effect of coexisting comorbid conditions in HF on worse clinical outcomes, higher symptom burden, poor medication adherence [1], and its relationship with reduced self-management ability [4, 16] shows the vital need for symptom management and medication adherence in HF patients with comorbidities [4, 8, 12, 17]. The significant effect of non-invasive telemonitoring on HF care-related costs, the incidence of acute non-fatal HF events [19], and the impact of telephone-based self-management programs on improving health awareness and symptom recognition is clear [20].

Some HF management interventions, such as educational counseling and exercise programs, have not affected QoL or echocardiographic parameters [12]. Moreover, interventions such as tele monitoring and nurse-driven health coaching telephone calls made no significant difference in readmission levels [6]. These studies, mainly focusing on educational counseling by the nurse or pharmacist, have used heterogeneous methods and have conflicting results [3, 11, 21]. Therefore, given the complexity of HF management and the need to treat the whole patient and improve measures of outcomes, multidisciplinary interventions are needed with a focus on the postdischarge phase [6, 12].

MMPs significantly affected the patients’ disease knowledge and QoL [22]. A systematic review and meta-analysis showed that multidisciplinary interventions have the best evidence for reducing the risk of HF readmissions for up to six months after discharge [23]. According to Ortiz-Bautista et al.(2019), using a nurse-led intervention significantly has improved perceived QoL and reduced hospital readmissions. Also, higher members of the program team and professional coordination were factors related to program success [21]. The results of a systematic review showed that in order to prevent 30-day readmission, the presence of a nurse as the coordinator of the care program is necessary[24]. However, another systematic review and meta-analysis showed that nurse-led multidisciplinary clinics have no advantage in reducing all-cause mortality and HF hospitalization [25]. Therefore, there are contradictory findings about MMPs [23].

Moreover, with a complex and inflexible structure and the primary goal of increasing survival and readmission [26], MMPs are mainly implemented in high-income countries [7]. Therefore, the binding effect of comorbidities and self-care behaviors on HF management is neglected [20, 27]. A study showed that despite the severity of their illness, paradoxically, HF patients with comorbidities such as diabetes mellitus, stroke, and CKD are less likely to be followed up by multidisciplinary clinics [28].

In many MMPs and clinical trials, comorbidities have been considered exclusion criteria, and data from such trials cannot be generalized [1, 29]. Therefore, there is a gap in the optimal management of comorbidities, unequal access to health services [27], and the need to assess medication adherence interventions in different healthcare systems [11]. Furthermore, there is a need to recruit comorbidities in future clinical trials [1] and specialized symptom management and polypharmacy in HF. This study aimed to examine the effects of a multidisciplinary management program (MMP) on symptom burden and medication adherence in HF patients with comorbidities.

## Methods

### Design

This study is a two-group randomized controlled clinical trial.

### Setting and participants

**Fig. 1:**
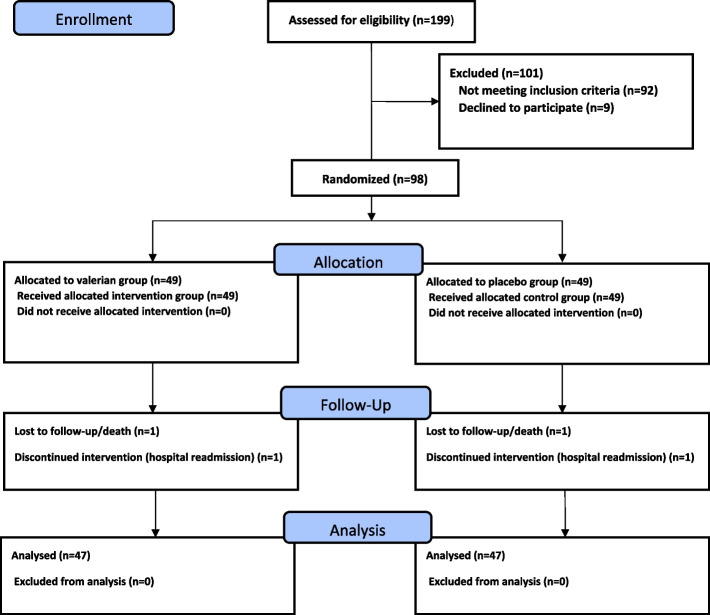
Flow diagram of the study

The study population included 199 patients with a primary diagnosis of HF exacerbation who were planned to be discharged after staying in a teaching hospital affiliated with Lorestan University of Medical Sciences in Iran. They were invited and screened for inclusion in the study by the first author between January 2019 and April 2020. Following eligibility screening, 101 patients were excluded for various reasons (Fig. 1).

The study inclusion criteria are as follows 30–80 years of age, admission with systolic/diastolic HF New York Heart Association (NYHA) class II to IV with left ventricular ejection fraction (LVEF) ≤ 50%, hospitalization due to typical HF-related signs and symptoms, with at least two cardiac comorbidities such as hypertension, coronary artery disease, or non-cardiac comorbidities such as diabetes mellitus, CKD, according to Charlson Comorbidity Index (CCI) [15], use of guideline-based medications during hospitalization, use of at least four drugs or more in the past three months, no changes in heart medications in the past month, with no debilitating auditory, visual, and speech impairments, no severe cognitive disorders and orientation to person, place and time, willingness to take part in the study, ability to communicate in Persian and access to a fixed or mobile phone.

Exclusion criteria were being transferred to a nursing home or palliative care center, survival of fewer than six months, alcohol/drug dependence, readmission during the study (due to dangerous arrhythmias, unstable hemodynamics, oncology emergencies), diagnosis of psychiatric disorders requiring active treatment, requiring dialysis, serious diseases such as severe liver failure or acute infectious diseases, requiring invasive interventions, and participation in other cardiac rehabilitation programs during the study.

### Sampling and estimation of the sample size

Eligible patients were first included in the study through non-probability consecutive sampling and then assigned to MMP or Usual Care (UC) groups using the stratified block random sampling method.

Based on a previous study and mean changes in the score of symptom burden as the secondary endpoint before and after a home-based care program [30], also using G-power 3.0.10 and estimating an average effect size of 0.40, the expected correlation in repeat measurements of 0.7, confidence interval of 95%, and test power of 0.80, the sample size was initially determined 42 persons per group, which was increased to 50 persons per MMP and UC groups, taking into account possible withdrawal of 20% during follow-up, making a total of 100 persons.

#### ***Randomization***

A cardiologist, before randomization, carried out medical history, physical examination, echocardiographic examination, and electrocardiography test. After collecting the baseline data and signing the informed consent form, eligible patients were assigned to MMP and UC groups by stratified block randomization performed by the statistician to match the two groups in terms of gender, NYHA class, and hypertension/diabetes mellitus. First, two gender categories (male and female) were formed. For each gender group, patients were subcategorized as having hypertension/diabetes mellitus (yes, no) and HF classes (II to IV) in blocks of four and allocation ratio of 1:1. They were randomly assigned to MMP and UC groups using a computer-generated code.

#### Blinding

A nurse with no role in the study encoded groups as A (intervention) and B (control), which was not disclosed by this independent nurse until data analysis ended. The data analyst, assessment nurse (data collector), and participants were blinded to the random allocation of groups. Blinding the interventionists was not possible due to the nature of the study.

#### Intervention

**Fig. 2:**
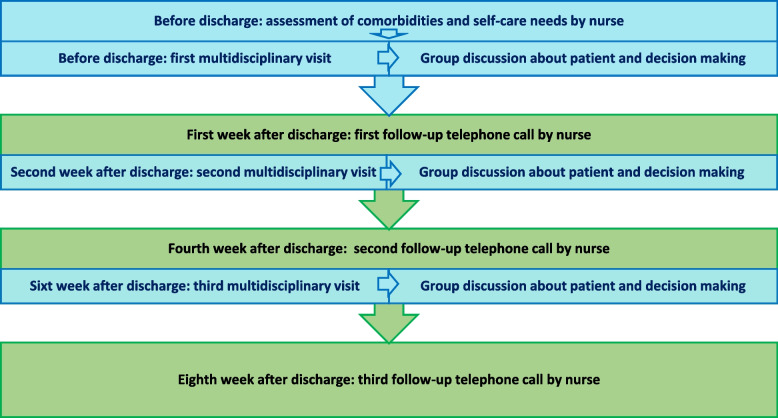
The specific structure of the multidisciplinary management program (MMP)

The intervention included a Nurse-led MMP focusing on three multi-professional visits, a training booklet, and three telephone follow-ups aiming at self-management or multi-symptom self-monitoring and multi-drug management over eight weeks (Fig. 2). The multi-professional management team included a cardiologist, a clinical pharmacist, a registered nurse, and a nutritionist. The registered nurse was a liaison between patients and the multi-professional management team.

The order of the multi-professional visits and telephone follow-ups was as follows:

##### Multi-Professional Visits

Each patient was visited three times. The first was at the time of patient discharge in the ward. The second and third visits were performed in the second and sixth weeks after discharge, respectively, in a secluded room in a cardiac clinic affiliated with the hospital. During the multi-professional visits, the responsibility of their members and their order were as follows:

- The first visit: Six to twelve hours before discharge, an experienced nurse in HF self-management arranged the time of the first multidisciplinary visit with the patient and multidisciplinary team after introducing themselves and presenting the manual of the disease management program and explaining its purpose to the patient. Before the visit, the nurse determined the patient’s comorbidity burden according to CCI to implement patient-oriented care. They also carried out an initial assessment of the patient’s care needs. The nurse reported the comorbidity status to other team members. Then, a cardiologist came to the patient's room for a clinical visit in the presence of a nurse and administered medications and performed other clinical assessments while explaining the patient’s current cardiac health status, taking into account the type and burden of comorbidities. After the cardiologist, a pharmacist reviewed medications administered by the cardiologist and consulted them regarding changing or adjusting medications, and new medication changes were implemented accordingly. Then, the pharmacist gave a speech to teach the patient how to take medications, interactions (drug, food, etc.), and manage their side effects in 10–15 min. Next, the nutritionist measured the body mass index (BMI) and cachexia associated with HF Nutrition counseling was carried out over 10–15 min by focusing on managing nutritional risk factors, a low-salt diet, recommending antioxidants, and paying attention to food labels. In the next step, the nurse trained the patients by defining how to manage comorbidities (according to the baseline assessments), identify HF's symptoms and adhere to medication regimen as well as methods to improve quality of life using the face-to-face method and simple figures for 20–30 min. At the end of the visit, the nurse gave patients an educational booklet titled "Living with Heart Failure" to reinforce and remind the intervention and reviewed the contents for over 5 to 10 min for patients and family caregivers.
- The second and third visits were similar to the first in coordination with the nurse and the order and duties of the team members, except that they were performed in the HF clinic for over 40 min. First, the nurse took history, especially regarding improving the previous symptoms and following the medication instructions, and controlled the patient's vital signs. Then, the physician performed a clinical examination and monitored the patient’s cardiac health, and if required, para clinical assessments and new medications were administered to the patient. Next, while reviewing previous and new drugs and consulting with the cardiologist, the pharmacist changed the medication prescriptions and provided the patient with medication counseling focused on comorbidity and polypharmacy. Nutrition counseling mainly focuses on checking lipid and sugar profiles, assessing new nutritional status, and helping the patient overcome barriers to healthy eating. The nurse's counseling focused on assessing symptom changes, managing new and multiple symptoms in comorbidities, and directing the patient to overcome obstacles to adherence to numerous medications.

At the end of the visits, a group meeting was held in a secluded room with the presence of the team professionals. New changes in the patient care program were implemented. If necessary, team members exchanged their views after the nurse reviewed the patient’s health status. Attempts were made in all multi-professional visits to respond to the patient's questions and involve family caregivers in the counseling sessions.

##### Telephone Follow-ups

Three home-based telephone follow-ups were performed for each patient by the multi-professional team nurse in the first, fourth, and eighth weeks following discharge, respectively, to discover new needs and ongoing self-management monitoring. First, previous training was reviewed and asked of the patient, self-management problems were assessed, and new recommendations were provided. Also, the patient was reminded to attend the clinic for multi-professional visits. This telephone contact lasted 10 to 15 min on average and occurred between 8 am and 8 pm as it suited the patients.

During these visits or phone calls, If the patient-reported worsening symptoms or team members detected problems with comorbidities, the team cardiologist referred the patient to other relevant specialists in coordination with the team nurse.

##### “Living with Heart Failure” Booklet

This booklet was compiled based on a literature review relating to HF management and comorbidities in HF [27, 31, 32]. The contents of this educational booklet included a definition of heart failure, symptoms, diagnosis, management, description and management of comorbidities, management of multiple symptoms, sleep hygiene, healthy nutrition, self-care skills, weight monitoring, polypharmacy management, management of medication side effects, management of cardiovascular risk factors and lifestyle adjustments, breathing exercises, annual vaccinations, sexual health, promotion of mental and social health, supportive therapies, clinic visits or consultation with the multidisciplinary team, and care with a holistic approach. The booklet was compiled in simple language, and its textual clarity was assessed. The validity of its contents was evaluated by the members of the same multi-professional team and the leading researcher.

The interventional nurse had 15 years of experience caring for cardiovascular patients and participated in self-management support and inter-professional communication workshops to manage the disease, facilitating multi-professional coordination and joint decision making. Also, several methods were used as fidelity-promoting strategies, including randomly recording participants’ voices in visits and telephone follow-ups and including them in discussions with the study team, plus a logbook for recording symptoms/medications, care plan, and objectives and content of each visit or telephone follow-up.

An educational booklet was given to the control group of patients receiving optimal medication therapy (such as ACEIs and diuretics) before discharge. If required, the patient was directed to the specialty office practice visits. But no regular clinical visits, telehealth monitoring, or telephone follow-ups were provided after discharge.

### Data collection and outcome measures

At baseline before discharge (T1), sociodemographic and clinical details of patients were collected through self-reports and electronic medical records. Sociodemographic and clinical data including age, gender, education, marital status, HF etiology, NYHA functional class, LVEF, duration of the disease, hospitalizations due to HF, HF medication history, medications used for comorbidities, BMI, vital signs, laboratory test results such as serum sodium and potassium, were collected through self-reports and electronic medical record.

Medication adherence as the primary outcome and symptom burden as the secondary outcome as well as the burden of comorbidities were measured using structured scales on three occasions: Baseline/before discharge (T1), week six (T2), and week eight (T3) after discharge. The scales were completed at the baseline (T1) through medical records (for comorbidity index) and in-person interviews and during the study (T2), and at the end of the study (T3) through telephone interviews. Assessments were carried out by a trained nurse independent from the intervention team.

Medication adherence was assessed using the Morisky Medication Adherence Scale (MMAS). MMAS contains eight items. The score on this scale ranges from 0 to 8 [33]. Adherence is considered low for scores greater than two, medium for scores one and two, and high for zero. For the original version of MMAS, internal consistency (Cronbach’s alpha reliability), sensitivity and specificity were 0.83, 0.93, and 0.53, respectively [34]. Many countries have validated this scale [35]. The present study confirmed its reliability with Cronbach’s alpha of 86%.

The Edmonton Symptom Assessment Scale (ESAS) assessed the symptom burden. ESAS is a valid and reliable tool for evaluating symptoms including pain, fatigue, dyspnea, appetite, vomiting, constipation, depression, anxiety, drowsiness, and feeling of well-being. According to the Visual Analog Scale (VAS), each mark is graded from zero (best situation) to 10 (worse situation) [30]. In the present study, Cronbach’s alpha of 0.85 confirmed the reliability of ESAS. Moreover, after determining its content validity by three cardiologists and four cardiac nursing teachers, the symptom of pain was changed to chest pain. Furthermore, two other symptoms associated with HF, including problems with consciousness and inability to activity, were added. In total, the severity of 12 signs was assessed. In the present study, symptom burden was graded according to the number of symptoms experienced. Having 1–4 symptoms was considered a “mild burden,” 4.1–8 symptoms as “medium burden,” and 8.1–12 as “severe burden.”

The CCI is a comorbidity assessment method in patients based on the International Classification of diseases diagnosis [15]. Diagnoses include the following 17 conditions if they exist in the patient's medical records: Stroke/transient ischemic attack (TIA), hypertension, myocardial infarction, congestive HF, peripheral vascular disease, dementia, diabetes mellitus with or without end-organ damage, chronic liver disease, COPD, peptic ulcer, depression, connective tissue disease, renal disease, cancer (leukemia, lymphoma, and tumor), hemiplegia, warfarin use, and human immunodeficiency virus (HIV) with or without acquired immunodeficiency syndrome (AIDS). An associated weight is assigned to each comorbidity (score of 1, 2, 3, or 6). The total weighted score is the final comorbidity index score [30]. In the present study, congestive HF was excluded. Also, based on the frequency of comorbidities, four grades were defined, including mild burden (2–3), medium (4–6), severe (7–9), and very intense comorbidity (over nine diseases).

### Statistical analysis

All continuous variables are expressed as mean ± standard deviation. Normality of data was assessed using Kolmogorov–Smirnov test. The baseline comparison between groups was performed using a student’s t-test for the normally distributed variables (LVED, BMI) and Chi-squared or Fisher’s exact test for nominal and categorical variables (reasons of hospitalization, e.g., age group, gender, etc.). The repeated-measures ANOVA was used to assess mean changes in medication adherence and symptom burden over three different occasions. Mean values of comorbidity burden on two events after discharge were compared using independent t-tests, and mean values of symptom burden and medication adherence on these two occasions were compared using independent t and paired t-tests. To adjust for confounding and underlying variables such as the number of medications, mixed models such as GEE with identity link function and the Friedman test were used if required. Data were analyzed in SPSS-19 at a significance level of *P* < 0.05.

## Results

### Baseline sociodemographic and clinical characteristics

Ninety-four patients (50% women and 50% men) completed the study. The patients’ mean age was 68.85 ± 1.46 years in the MMP group (intervention) and 71.38 ± 2.03 years in UC (control). Most participants were married (91.5%), with low literacy (72.3%), HF history ≥ 5 years (52.1%), NYHA functional class 3 (39.3%), LVEF < 40% (80.8%) and ischemic etiology (82.9%). Over half of the patients had a hospitalization history ≥ 4 times (55.3%) for HF Most medications administered for HF included ACEI/ARB (86.1%), and more than half of them used 2–4 medications for their comorbidities (53.1%). Regarding BMI, both groups were considered overweight, but vital signs and paraclinical markers were almost normal (Table 1). At baseline, no significant difference was observed between the two groups in demographic-clinical characteristics, including age, gender, education, HF etiology, NYHA functional class, LVEF, frequency of hospitalization, and use of HF medications clinical examination and laboratory assessments (P > 0.05). There were significant differences between the two groups regarding marital status, diagnosis time, and the number of medications taken for comorbidities (Table 1).

**Table 1:** Baseline characteristics of the study patients (*n* = 94)

Characteristic	MMP (*n* = 47)	UC (*n* = 47)	*P*-value
Age group (years), n (%)			0.328 ^a^
< 65	27 (57.4)	22 (46.8)	
≥ 65	20 (42.6)	25 (53.2)	
Gender, no. (%)			0.680 ^a^
Female	22 (46.8)	25 (53.2)	
Male	25 (53.2)	22 (46.8)	
Marital status, no. (%)			0.013 ^a^
Married	39 (82.9)	47 (100)	
Widowed/ Divorced	8 (17.1)	0 (0)	
Education, no. (%)			0.163 ^a^
Primary school	36 (76.6)	32 (68.1)	
Middle school	7 (14.9)	9 (19.1)	
Diploma or higher	4 (8.5)	6 (12.8)	
HF etiology, no. (%)			0.785 ^a^
Ischemic	40 (85.1)	38 (80.9)	
Nonischemic	7 (14.9)	9 (19.1)	
Time of diagnosis (years), n (%)			< 0.001 ^a^
< 5	13 (27.7)	32 (68.1)	
≥ 5	34 (72.3)	15 (31.9)	
HF hospitalization, no. (%)			0.230 ^a^
≤ 3	20 (42.6)	22 (46.8)	
≥ 4	27 (57.4)	25 (53.2)	
NYHA class, no. (%)			0.971 ^a^
II	15 (31.9)	16 (34.1)	
III	19 (40.4)	18 (38.2)	
IV	13 (27.7)	13 (27.7)	
LVEF, no. (%)			0.432 ^a^
< 40%	40 (85.1)	36 (76.6)	
≥ 40%	7 (14.9)	11 (23.4)	
HF Medication Prescribed, Yes, no. (%)			
ACEi/ARB	40 (85.1)	41 (87.2)	0.990 ^a^
Beta-blocker	28 (59.6)	21 (44.7)	0.215 ^a^
Diuretics ^c^	23 (48.9)	21 (44.7)	0.836 ^a^
Calcium channel blocker	6 (12.8)	7 (14.9)	0.331 ^a^
Digitalis	14 (29.8)	17 (36.2)	0.661 ^a^
Comorbidities medication, Yes, no. (%)			< 0.001 ^a^
2–4	10 (21.3)	40 (85.1)	
5–7	19 (40.4)	6 (12.8)	
8–10	18 (38.2)	1 (2.1)	
Clinical examination, mean (SD.)			
BMI, kg/m^2^	27.0 ± 4/5	25.3 ± 4/4	0.069 ^b^
SBP, mmHg	127.5 ± 25/9	127.0 ± 25.1	0.937 ^b^
DBP, mmHg	78.6 ± 11.3	80.1 ± 15.0	0.580 ^b^
Heart rate, bpm	78.8 ± 2.6	80.1 ± 1.9	0.522 ^b^
Laboratory value, mean (SD.)			
Serum sodium, mEq	140.1 ± 3.9	140.5 ± 3.5	0.639 ^b^
Serum potassium, mEq	4.1 ± 0.5	4.0 ± 0.4	0.684 ^b^
Serum creatinine, mg/dL	1.2 ± 0.4	1.3 ± 0.3	0.235 ^b^
Hemoglobin, g/L	13.3 ± 1.9	13.4 ± 1.7	0.787 ^b^

MMP, multidisciplinary management program; UC, usual care; SD, standard deviation; HF, heart failure; ACEi, angiotensin-converting enzyme inhibitor; ARB, angiotensin receptor blocker; NYHA, New York Heart Association; LVEF, left ventricular ejection fraction; BMI, body mass index; SBP, systolic blood pressure; DBP, diastolic blood pressure

^c^ Includes loop diuretics, thiazides, and aldosterone antagonists

^a^ Pearson Chi-squared test

^b^ Independent sample t-test

#### Comorbidities

At baseline, the most frequent comorbidities in the MMP group included myocardial infarction (82.9%), hypertension (80.8%), peptic ulcer (53.2%), and depression (51.1%). Likewise, the most frequent comorbidities in the UC group included myocardial infarction and hypertension (72.3%), peptic ulcer (51.1%), and depression (34.1%). HIV was not reported in any of these groups (Table 2). However, as **Appendix ****1** shows, most patients (between 46 to 53%) had a medium burden of comorbidities. No significant difference was found between MMP and UC groups in terms of different degrees of comorbidities (low, medium, severe, and very severe) on the three occasions: baseline, Time 2, and Time 3 (*P* > 0.05).

**Table 2:** The two groups' absolute and relative frequency of the CCI at three time-points

Variables	Time 1 ^*^		Time 2 ^*^		Time 3 ^*^	
Comorbidities profile	MMP	UC	MMP	UC	MMP	UC
Hypertension, no. (%)	38 (80.8)	34 (72.3)	40 (85.1)	35 (74.5)	42 (89.4)	35 (74.5)
Myocardial infarction, no. (%)	39 (82.9)	34 (72.3)	41 (87.2)	36 (76.6)	37 (78.7)	35 (74.5)
Diabetes mellitus, no. (%)	17 (36.1)	13 (27.7)	17 (36.1)	13 (27.7)	17 (36.1)	13 (27.7)
COPD, no. (%)	10 (21.3)	13 (27.7)	10 (21.3)	13 (27.7)	10 (21.3)	13 (27.7)
Renal disease, no. (%)	14 (29.8)	11 (23.4)	16 (34.0)	12 (25.5)	16 (34.0)	13 (27.7)
Liver disease, no. (%)	0 (0)	1 (2.1)	0 (0)	1 (2.1)	0 (0)	1 (2.1)
Peptic ulcers, no. (%)	25 (53.2)	24 (51.1)	34 (72.3)	32 (68.1)	36 (76.6)	32 (68.1)
Connective tissue disease, no. (%)	7 (14.9)	4 (8.5)	6 (12.8)	4 (8.5)	5 (10.6)	4 (8.5)
Peripheral vascular disease, no. (%)	3 (6.4)	5 (10.6)	3 (6.4)	3 (6.4)	5 (10.6)	3 (6.4)
Cancer, no. (%)	8 (17.1)	3 (6.4)	8 (17.1)	3 (6.4)	8 (17.1)	3 (6.4)
Hemiplegia, no. (%)	6 (12.8)	5 (10.6)	3 (6.4)	5 (10.6)	3 (6.4)	5 (10.6)
Stroke/ TIA, no. (%)	4 (8.5)	8 (17.1)	5 (10.6)	8 (17.1)	5 (10.6)	9 (19.1)
Dementia, no. (%)	3 (6.4)	1 (2.1)	1 (2.1)	2 (4.2)	1 (2.1)	1 (2.1)
Depression, no. (%)	24 (51.1)	16 (34.1)	24 (51.1)	24 (51.1)	26 (55.3)	17 (36.1)
HIV, no. (%)	0 (0)	0 (0)	0 (0)	0 (0)	0 (0)	0 (0)
Taking warfarin, no. (%)	3 (6.4)	5 (10.6)	3 (6.4)	5 (10.6)	2 (4.2)	4 (8.5)

CCI, Charlson comorbidity index; COPD, chronic obstructive pulmonary disease; TIA, transient ischemic attack; HIV, human immunodeficiency virus

^*^ Time 1 (baseline/pre-discharge), Time 2 (the sixth week, post-discharge), Time 3 (the eighth week, post discharge)

### Medication adherence

In the MMP group, the mean score of medication adherence significantly changed over time, such that the mean score of medication adherence from Time 1 to Time 2 and from Time 2 to Time 3 significantly improved compared to the UC group (*P* < 0.001). The interactive effect of time and group showed a significant difference between mean changes in medication adherence scores on different occasions (*P* < 0.001), such that the increase in medication adherence in the MMP group from Time 1 to Time 2 and from Time 2 to Time 3 was significant compared to the control group (*P* < 0.001). Compared to the MMP group, a significant reduction in medication adherence was observed in the UC group (*P* = 0.017) (Table 3).

**Table 3:** Comparison of the ESAS dimensions, ESAS total, and the MMAS total by groups over three-time points

Variables	Group (each group *n* = 47)	Time 1 ^*^	Time 2 ^*^	Time 3 ^*^	*P*-value (Time1 vs Time 2	*P*-value (Time 2 vs. Time 3)	*P*-value within group	*P*-value Interaction effect Time × group ^a^
Symptom burden dimensions, mean ± SD								
Chest pain	**MMP**	6.76 ± 1.73	5.02 ± 1.62	4.23 ± 1.34	< 0.001	0.016	< 0.001	< 0.001
	**UC**	6.27 ± 1.79	6.65 ± 1.10	6.91 ± 1.34	0.146	0.204	0.120	
	**P-value** **between**	< 0.001 < 0.001						
Fatigue	**MMP**	7.00 ± 1.25	5.85 ± 1.38	5.00 ± 1.78	< 0.001	0.005	< 0.001	< 0.001
	**UC**	6.57 ± 1.47	6.87 ± 1.19	6.85 ± 1.30	0.221	0.929	0.430	
	**P-value** **between**	< 0.001 0.028						
Drowsiness	**MMP**	6.00 ± 1.51	5.25 ± 1.52	4.12 ± 1.97	0.002	< 0.001	< 0.001	< 0.001
	**UC**	5.74 ± 1.67	6.08 ± 1.52	6.17 ± 1.18	0.179	0.721	0.256	
	**P-value** **between**	0.002 0.002						
Nausea	**MMP**	5.57 ± 1.93	4.61 ± 1.29	3.53 ± 1.12	< 0.001	< 0.001	< 0.001	< 0.001
	**UC**	5.34 ± 1.72	5.68 ± 1.14	5.65 ± 1.41	0.262	0.934	0.508	
	**P-value** **between**	0.002 0.002						
Loss of appetite	**MMP**	5.34 ± 1.95	4.19 ± 1.52	3.31 ± 1.57	< 0.001	0.022	< 0.001	< 0.001
	**UC**	5.53 ± 1.93	6.17 ± 1.74	6.17 ± 1.68	0.160	0.990	0.290	
	**P-value** **between**	< 0.001 0.040						
Constipation	**MMP**	6.21 ± 1.92	7.08 ± 0.95	4.95 ± 1.86	0.319	0.001	< 0.001	< 0.001
	**UC**	5.51 ± 1.50	5.95 ± 1.45	7.95 ± 1.86	0.023	0.001	0.021	
	**P-value** **between**	0.021 0.002						
Dyspnea	**MMP**	6.53 ± 1.75	5.14 ± 1.65	4.48 ± 1.41	< 0.001	0.008	< 0.001	< 0.001
	**UC**	6.29 ± 1.73	6.34 ± 1.55	6.59 ± 1.67	0.877	0.345	0.544	
	**P-value** **between**	< 0.001 0.01**2**						
Depression	**MMP**	5.76 ± 1.68	5.19 ± 1.54	5.00 ± 1.68	0.040	0.509	0.037	0.006
	**UC**	5.40 ± 1.86	5.93 ± 1.45	6.14 ± 1.48	0.090	0.323	0.088	
	**P-value** **between**	0.009 0.262						
Anxiety	**MMP**	6.59 ± 1.45	5.74 ± 1.30	4.70 ± 1.66	< 0.001	0.001	< 0.001	< 0.001
	**UC**	5.85 ± 1.60	6.31 ± 1.27	6.36 ± 1.35	0.102	0.811	0.234	
	**P-value** **between**	< 0.001 0.003						
Feelings of well-being	**MMP**	6.23 ± 1.23	4.76 ± 1.27	4.42 ± 1.42	< 0.001	0.195	< 0.001	< 0.001
	**UC**	5.55 ± 1.28	6.29 ± 1.33	6.34 ± 1.49	0.015	0.855	0.029	
	**P-value** **between**	< 0.001 0.273						
Inability to Activity	**MMP**	5.80 ± 1.65	5.02 ± 1.49	4.24 ± 1.63	< 0.001	0.040	< 0.001	< 0.001
	**UC**	5.80 ± 1.82	6.85 ± 1.45	7.02 ± 1.43	< 0.001	0.400	< 0.001	
	**P-value** **between**	< 0.001 0.044						
Problems with consciousness	**MMP**	4.55 ± 1.51	3.27 ± 0.79	2.77 ± 1.19	< 0.001	0.053	< 0.001	< 0.001
	**UC**	4.02 ± 1.52	4.17 ± 1.22	3.85 ± 1.39	0.582	0.190	0.409	
	**P-value** **between**	< 0.001 0.787						
Total symptom burden	**MMP**	6.03 ± 1.00	5.00 ± 0.91	4.27 ± 0.89	< 0.001	< 0.001	< 0.001	< 0.001
	**UC**	5.71 ± 1.02	6.20 ± 0.87	6.27 ± 1.04	0.004	0.648	0.002	
	**P-value** **between**	< 0.001 < 0.001						
**Total medication adherence,** mean ± SD	**MMP**	43.30 ± 13.20	23.90 ± 13.67	18.35 ± 8.56	< 0.001	0.013	< 0.001	< 0.001
	**UC**	43.30 ± 14.00	50.00 ± 14.25	50.79 ± 14.90	0.014	0.755	0.017	
	**P-value** **between**	< 0.001 < 0.001						

MMP, multidisciplinary management program; UC, usual care; SD, standard deviation; ESAS, Edmonton symptom assessment scale; MMAS, Morisky medication adherence scale

^*^ Time 1 (baseline/pre discharge), Time 2 (the sixth week, post-discharge), Time 3 (the eighth week, post-discharge)

^a^ Repeated measures analysis of variance

The difference between absolute or relative frequency was significant between the two groups for all medication adherence scores on two occasions after the intervention (*P* < 0.001) (**Appendix ****2**).

#### Symptom burden

In the MMP group, there were significant differences in the mean score of severity of chest pain, fatigue, drowsiness, nausea, lack of appetite, dyspnea, and anxiety over time, such that the mean score of these seven symptoms significantly reduced from Time 1 to Time 2 and from Time 2 to Time 3 compared to the UC group (*P* < 0.05). The interactive effect of time and group showed a significant difference in mean changes of scores of all symptoms and total score of the two groups on different occasions (*P* = 0.006 to *P* < 0.001). A significant reduction in the severity of symptoms was observed in the MMP group from Time1 to Time 2 and from Time 2 to Time 3 in all symptoms except for problems with consciousness, feeling of well-being, and depression compared to the UC group (*P* < 0.05) (Table 3).

There was a significant difference between the two groups in the absolute or relative frequency of all grades of symptom burden and on two occasions after the intervention (*P* < 0.001) (**Appendix ****2**).

## Discussion

Considering patients with HF and comorbidities, compared to the usual care, MMP can improve medication adherence and reduce symptom burden in a particular patient population, who had less benefited from such interventions in previous studies.

The frequency of comorbidities found in the present study is somewhat different from other studies. In other studies, hyperlipidemia, anemia [36] or renal failure, diabetes, and atrial fibrillation [2, 37] were identified as the third or fourth comorbidities, but in the current study, these ranks were assigned to ulcerative colitis and depression. The burden and frequency of comorbidities can change the biological response to a trial therapy or risk–benefit balance and the ability to adhere to a self-care program [1]. Therefore, data from other trials cannot be generalized to our daily clinical practice and highlighted the need to manage more familiar patterns of comorbidities such as psychological and gastrointestinal disorders for patients with HF in the present study.

Vulnerable individuals with comorbidities are less likely to receive optimal evidence-based HF treatment [21]. However, the effect of intervention in steadily improving medication adherence was determined in the present study. In a cohort study, implementing a multidisciplinary cardiology service in a low-income setting was associated with increased guideline-directed medical therapy [38]. In their meta-analysis study, Parajuli et al. showed that pharmacist-involved multidisciplinary teams significantly reduce HF hospitalization (28%) and all-cause hospitalizations but have no effect on HF mortality and all-cause mortality [39].

These results reflect those of Dijkstra et al. (2021), who also found that supportive nursing interventions positively affect medication adherence. In this qualitative study, the participants were satisfied with the intended intervention but wanted to receive more services [40]. Also, These results are consistent with data obtained in Gwadry-sridhar et al. (2005) and Heloue et al.(2016). Gwadry-sridhar et al. (2005) suggested that the in-hospital multidisciplinary educational intervention in HF patients improves their medication adherence [41]. Heloue et al. (2016) also investigated the effect of a multidisciplinary self-care management program on the adherence of renal patients to antihypertensive treatment, and they reached the same results as the present study [42].

Mixed or relatively low effect sizes have been reported for many interventions to improve medication adherence. However, the effectiveness of these interventions increases when the focus is only on changing medication adherence and seeking to change patients’ behavior rather than healthcare providers’ behavior [10, 11].

Medication adherence is difficult in HF patients and requires macro-cognitive processes such as sense-making, coordination, planning, monitoring, and decision-making [33]. Moreover, discharge and postdischarge are challenging and confusing experiences for HF patients [13, 43]. Many instances of medication training through text-based tools and didactic tools such as brochures and medication flyers are not learned. Therefore, optimal medication management, especially in patients with comorbidities, requires such approaches as skill-building and debriefing [12], integration of family-focused and literacy-sensitive strategies [44], use of communication tools such as pictograms to increase visual attention [45], provision of products with innovative packaging (like blister packs), use of fixed combination drugs and simplification of drug regimen [13].

Medication adherence strongly correlates with symptom management [8]. Therefore, a reduction in symptom burden after improving medication adherence was to be expected in the present study. Despite more significant symptom relief in our intervention group, the improvement in depression, feeling of well-being, and problems with consciousness were not substantial. It appears that the progress of these symptoms requires a longer follow-up time and more specialized interventions. A systematic review showed that the most beneficial of multidisciplinary clinics are in reducing all-cause mortality and HF hospitalization for patients in unstable conditions who had been recently hospitalized and had at least three months of follow-up visits [25]. However, the pathophysiological role of comorbidities should not be overlooked in the lack of improvement in all symptoms. Comorbidities, by causing sarcopenia, cachexia development, and amplifying catabolic processes, as well as increasing oxidative stress, activation of the neuroendocrine system and adrenergic system alterations and brain endothelium, entail the risk of exacerbating HF symptoms such as impaired daily activity, cognitive impairment, and reduced energy [31]. Patients with HF and comorbidities experience higher stress levels and struggle with illness. Therefore, they are likely to make different decisions about symptom management compared to those without comorbidity [46].

In the present study, the burden of medium and high severity symptoms gradually reduced over time in the intervention group compared to the control group. In agreement with this finding, in their study, Ng and Wong showed the effect of a palliative home-based HF program overtime on improving quality of life and symptoms such as dyspnea, tiredness, and total symptom burden score [30]. Symptom management as a self-care behavior is affected by knowledge of HF, correct perception and interpretation, and monitoring symptoms [14]. In the present study, the multidisciplinary team tried to teach patients to record their signs and symptoms on a patient chart after discharge. Biological indicators and symptoms were interpreted for patients during face-to-face visits. If abnormalities were reported, recommendations for managing HF-specific symptoms were provided in the patient's native language. Therefore, these potential mechanisms, together with an excellent nurse-patient relationship and multidisciplinary rounds, can be effective in the effectiveness of the present intervention.

The present study had several limitations. This study was conducted in one center and for a short period of two months. Given that symptom burden is a fluid concept and changes in the course of HF, longer interventions and assessment of outcomes during follow-up are needed. Assessment of medication adherence based on self-report is an indirect assessment. However, direct measures such as detecting drugs in blood or urine or detecting a biomarker will also be helpful. Moreover, some clinical experts were not used in the present intervention team. Given the profile of comorbidities in any society, there seems to be a need for more specialists such as psychologists, physiotherapists, internists, nephrologists, and pulmonologists to manage HF comprehensively.

## Conclusion

The present study is in line with the importance of shifting the HF management paradigm from usual care to identification and optimal management of comorbidities. The results suggest that a nurse-led multidisciplinary management program is a promising strategy for symptom management and medication adherence in HF patients with comorbidities. Given the mild burden and frequency of comorbidities, and interactive and reciprocal symptoms [15], there is a need for tailoring interventions with a patient-centric disease profile, especially for the cluster of neuropsychological symptoms in HF. Moreover, ongoing medication adherence and symptom management improvement require using digital solutions or smartphone and software applications in multidisciplinary management programs.

## Supplementary Information

**Additional file1.**

## Data Availability

The datasets generated and/or analyzed during the current study are not publicly available due to confidentiality but are available from the corresponding author on reasonable request.

## Abbreviations

HF: Heart Failure
MMP: Multidisciplinary Management Program
MMAS: Morisky Medication Adherence Scale
ESAS: Edmonton Symptom Assessment Scale
MI: Myocardial Infarction
Qol: Quality of Life
LVEF: Left Ventricular Ejection Fraction ()
CKD: Chronic Kidney Disease
NYHA: New York Heart Association
CCI: Charlson Comorbidity Index
UC: Usual Care
BMI: Body Mass Index
VAS: Visual Analog Scale
COPD: Chronic obstructive pulmonary disease
TIA: Transient Ischemic Attack
AIDS: Acquired Immunodeficiency Syndrome
HIV: Human Immunodeficiency Virus
