# Supplementary material for: Effects of a multidisciplinary management program on symptom burden and medication adherence in heart failure patients with comorbidities: A randomized controlled trial
Source: BMC Nurs. 2022 Dec 7;21:346. doi: 10.1186/s12912-022-01130-7 (PMC9727875; doi:10.1186/s12912-022-01130-7)
Supplement: Supplementary file 1 — Additional file1. [file 12912_2022_1130_MOESM1_ESM.docx]

Appendix 1. Comparison of the frequency of comorbidities burden in the two groups, at 3 time-points.

| **Variables** | **Time 1 ^*^** | | **Time 2 ^*^** | | **Time 3 ^*^** | |
| --- | --- | --- | --- | --- | --- | --- |
| **Comorbidities burden** | **MMP** (n=47) | **UC** (n=47) | **MMP** (n=47) | **UC** (n=47) | **MMP** (n=47) | **UC** (n=47) |
| Mild (2-3) | 7 (14.9) | 6 (12.8) | 1 (2.1) | 3 (6.4) | 0 (0) | 5 (10.6) |
| Moderate (4-6) | 22 (46.8) | 23 (48.9) | 22 (46.8) | 25 (53.2) | 24 (51.1) | 22 (46.8) |
| Sever (7-9) | 11 (23.4) | 13 (27.7) | 20 (42.6) | 15 (31.9) | 20 (42.6) | 16 (34.1) |
| Very sever (>9) | 7 (14.9) | 5 (10.6) | 4 (8.5) | 4 (8.5) | 3 (6.4) | 4 (8.5) |
| **Test ^a^ p-value**  **between group** | 0.897 | | 0.592 | | 0.129 | |

MMP, multidisciplinary management program; UC, usual care.

^*^ Time 1 (baseline/pre-discharge), Time 2 (the sixth week, post-discharge), Time 3 (the eighth week, post-discharge),

^a^ Chi-Square

Appendix 2. Comparison of the frequency of symptom burden grades and medication adherence scores in the two groups at 3 time-points.

| **Variables** | **Time 1 ^*^** | | **Time 2 ^*^** | | **Time 3 ^*^** | |
| --- | --- | --- | --- | --- | --- | --- |
| **Symptom burden grade, n (%)** | MMP (n=47) | **UC** (n=47) | MMP (n=47) | **UC** (n=47) | **MMP** (n=47) | **UC** (n=47) |
| Mild (1-4), n (%) | 1 (2.1) | 3 (6.4) | 7 (14.9) | 0 (0) | 23 (48.9) | 0 (0) |
| Moderate (4.1-8), n (%) | 38 (80.9) | 41 (87.2) | 40 (85.1) | 37 (78.7) | 24 (51.1) | 36 (76.6) |
| Sever (8.1-12), n (%) | 8 (17.1) | 3 (6.4) | 0 (0.0) | 10 (21.3) | 0 (0) | 11 (23.4) |
| **Test ^a^ p-value**  **between group** | 0.184 | | <0.001 | | <0.001 | |
| **Medication adherence score, n (%)** |  | |  | |  | |
| Low, n (%) | 1 (2.1) | 4 (8.5) | 0 (0) | 5 (10.6) | 0 (0) | 7 (14.9) |
| Medium, n (%) | 37 (78.7) | 31 (66.0) | 11 (23.4) | 37 (78.7) | 3 (6.4) | 34 (72.3) |
| High, n (%) | 9 (19.1) | 12 (25.5) | 36 (76.6) | 5 (10.6) | 44 (93.6) | 6 (12.8) |
| **Test ^a^ p-value**  **between group** | 0.252 | | <0.001 | | <0.001 | |

MMP, multidisciplinary management program; UC, usual care.

^*^ Time 1 (baseline/pre discharge), Time 2 (the sixth week, post-discharge), Time 3 (the eighth week, post-discharge).

^a^ Chi-square.
